# Supplementary material for: Performance evaluation of flexible macrocycle docking in AutoDock
Source: QRB Discov. 2022 Oct 17;3:e18. doi: 10.1017/qrd.2022.18 (PMC10392634; doi:10.1017/qrd.2022.18)
Supplement: Supplementary file 1 [file S2633289222000187sup001.docx]

# Supporting Information

Table S1. Representative set of complexes containing macrocyclic ligands.

| Entry ID | Resolution (Å) | Accession Code(s) |
| --- | --- | --- |
| 3KI1 | 1.43 | Q5EK40 |
| 6VCT | 1.94 | U3N5X4 |
| 5M4D | 1.93 | N6UXY4 |
| 2WEA | 1.25 | P00798 |
| 6RYP | 2.3 | Q6GHN9 |
| 6M4P | 2.3 | A0A286SBY7 |
| 6DIT | 1.789 | A0A0B4WYC6 |
| 6BBX | 2.2 | A0A125SA29 |
| 5EG4 | 1.32 | P00760 |
| 3ZWY | 2.4 | P29241 |
| 2PFG | 1.54 | P16152 |
| 6Z53 | 1.65 | P49761 |
| 4W9Q | 1.08 | Q13451 |
| 4QHC | 1.899 | P9WKD3 |
| 3ABA | 1.8 | Q93H81 |
| 2CD8 | 1.7 | O87605 |
| 1RQ9 | 2.6 | Q5RTL1 |
| 6Z5E | 1.5 | Q8TF76 |
| 6RCF | 1.1 | Q8N8S7 |
| 6MGO | 2.2 | P68135 |
| 6LOX | 3.2 | O94925 |
| 6G4Y | 2.65 | Q9WUL6 |
| 5T8O | 2.41 | Q9WUL6 |
| 4Z7M | 1.43 | P0AE18 |
| 4G9P | 1.55 | Q72H18 |
| 3EKS | 1.8 | P10987 |
| 2WER | 1.6 | P02829 |
| 2IWS | 2.7 | P02829 |
| 7UPJ | 2 | P03367 |
| 6A05 | 2.2 | B8XX90 |
| 5QTW | 2.12 | P03951 |
| 5QQP | 2.08 | P03951 |
| 4HAI | 2.55 | P34913 |
| 3OWN | 2 | P00797 |
| 3DJK | 1 | P03367 |
| 2ASP | 1.64 | P68135 |
| 1XA5 | 2.12 | P62157 |
| 1FQ5 | 2.4 | P07267 |
| 6LKA | 2.033 | A9XG43 |
| 5QTT | 2.23 | P03951 |
| 4ZBF | 2.2 | Q07820 |
| 6VVS | 3.112 | A0QSL8 |
|  |  | P60281 |
|  |  | A0QS66 |
|  |  | A0QWT1 |
|  |  | A0QW02 |
|  |  | A0QZ11 |
| 6R9U | 1.26 | P30405 |
| 6NV7 | 2.132 | P56817 |
| 5QDB | 2.1 | P56817 |
| 5QCO | 2.7 | P56817 |
| 5EQI | 3.002 | P11166 |
| 4DH0 | 2.1 | P62942 |
| 3FPD | 2.4 | Q9H9B1 |
| 1TVR | 3 | P03366 |
|  |  | P03366 |
| 1FKI | 2.2 | P62942 |
| 6W6D | 1.91 | Q96LA8 |
| 6NZT | 1.4 | S4UY05 |
| 5VP9 | 1.859 | P26664 |
| 5QOV | 1.65 | Q8IU60 |
| 5J2T | 2.2 | P81947 |
|  |  | Q6B856 |
|  |  | P63043 |
|  |  | E1BQ43 |
| 5GRM | 1.55 | F1M391 |
| 3C49 | 2.8 | Q9Y6F1 |
| 2IIT | 2.35 | P27487 |
| 4U01 | 2.8 | F0UY39 |
|  |  | Q9WMX2 |
| 4HLE | 2.78 | P48736 |
| 3R7R | 2.9 | P48736 |
| 3OVE | 1.82 | P62993 |
| 5TA4 | 1.5 | P62937 |
| 5L7H | 1.84 | P08235 |
|  |  | Q15788 |
| 5HI5 | 1.8 | Q16552 |
| 5HI4 | 1.8 | Q16552 |
| 3VHD | 1.52 | P07900 |
| 2P4N | 9 | P33176 |
|  |  | P02550 |
|  |  | Q6B856 |
| 1NM6 | 1.8 | P00734 |
|  |  | P28504 |
| 1NT1 | 2 | P00734 |
|  |  | P28504 |
| 1RE0 | 2.4 | P84077 |
|  |  | P47102 |
| 5BU3 | 1.897 | K7QVW7 |
| 4CP3 | 2.3 | P41182 |
| 1XZ0 | 2.8 | P06126 |
|  |  | P61769 |
| 3ZYA | 1.9 | Q16539 |
| 3K9X | 1.9 | P00742 |
|  |  | P00742 |
| 6KK4 | 1.74 | Q32ZE1 |
|  |  | Q32ZE1 |
| 5QDC | 2.1 | P56817 |
| 4KE0 | 2.3 | P56817 |
| 2WF3 | 2.08 | P56817 |
| 3C11 | 1.6 | P02829 |
| 6FII | 2.405 | P81947 |
|  |  | Q6B856 |
|  |  | P63043 |
|  |  | E1BQ43 |
| 5ICK | 2.47 | Q96RI1 |
|  |  | Q15596 |
| 5EOL | 2.2 | P11309 |
| 6XP6 | 2.4 | P01909 |
|  |  | Q5Y7D3 |
| 1UU3 | 1.7 | O15530 |
| 6VKK | 2.1 | P09874 |
| 2FAP | 2.2 | P62942 |
|  |  | P42345 |
| 1O46 | 2 | P12931 |

| PDB ID | Resolution (Å) |
| --- | --- |
| 1fki | 2.2 |
| 1fq5 | 2.4 |
| 1nm6 | 1.8 |
| 1nt1 | 2 |
| 1o46 | 2 |
| 1re0 | 2.4 |
| 1rq9 | 2.6 |
| 1tvr | 3 |
| 1uu3 | 1.7 |
| 1xa5 | 2.12 |
| 1xz0 | 2.8 |
| 2asp | 1.64 |
| 2cd8 | 1.7 |
| 2fap | 2.2 |
| 2iit | 2.35 |
| 2iws | 2.7 |
| 2p4n | 9 |
| 2pfg | 1.54 |
| 2wea | 1.25 |
| 2wer | 1.6 |
| 2wf3 | 2.08 |
| 3aba | 1.8 |
| 3c11 | 1.6 |
| 3c49 | 2.8 |
| 3djk | 1 |
| 3eks | 1.8 |
| 3fpd | 2.4 |
| 3k9x | 1.9 |
| 3ki1 | 1.43 |
| 3ove | 1.82 |
| 3own | 2 |
| 3r7r | 2.9 |
| 3vhd | 1.52 |
| 3zwy | 2.4 |
| 3zya | 1.9 |
| 4cp3 | 2.3 |
| 4dh0 | 2.1 |
| 4g9p | 1.55 |
| 4hai | 2.55 |
| 4hle | 2.78 |
| 4ke0 | 2.3 |
| 4qhc | 1.899 |
| 4u01 | 2.8 |
| 4w9q | 1.08 |
| 4z7m | 1.43 |
| 4zbf | 2.2 |
| 5bu3 | 1.897 |
| 5eg4 | 1.32 |
| 5eol | 2.2 |
| 5eqi | 3.002 |
| 5grm | 1.55 |
| 5hi4 | 1.8 |
| 5hi5 | 1.8 |
| 5ick | 2.47 |
| 5j2t | 2.2 |
| 5l7h | 1.84 |
| 5m4d | 1.93 |
| 5qco | 2.7 |
| 5qdb | 2.1 |
| 5qdc | 2.1 |
| 5qov | 1.65 |
| 5qqp | 2.08 |
| 5qtt | 2.23 |
| 5qtw | 2.12 |
| 5t8o | 2.41 |
| 5ta4 | 1.5 |
| 5vp9 | 1.859 |
| 6a05 | 2.2 |
| 6bbx | 2.2 |
| 6dit | 1.789 |
| 6fii | 2.405 |
| 6g4y | 2.65 |
| 6kk4 | 1.74 |
| 6lka | 2.033 |
| 6lox | 3.2 |
| 6m4p | 2.3 |
| 6mgo | 2.2 |
| 6nv7 | 2.132 |
| 6nzt | 1.4 |
| 6qiq | 2.519 |
| 6r9u | 1.26 |
| 6rcf | 1.1 |
| 6ryp | 2.3 |
| 6vct | 1.94 |
| 6vkk | 2.1 |
| 6vvs | 3.112 |
| 6w6d | 1.91 |
| 6z53 | 1.65 |
| 6z5e | 1.5 |
| 7upj | 2 |

Table S2. Complexes containing conformationally constrained macrocyclic peptides of therapeutic relevance.

| PDB ID | Resolution (Å) | UNIPROT ID |
| --- | --- | --- |
| 7NRF | 2.2 | P0A942 |
| 3S04 | 2.44 | P00803 |
| 4CPW | 1.7 | P03366 |
| 1B6P | 2 | P03369 |
| 1B6J | 1.85 | P03369 |
| 1RRV | 2 | Q9AFC7 |
